# Endothelial sprouting, proliferation, or senescence: tipping the balance from physiology to pathology

**DOI:** 10.1007/s00018-020-03664-y

**Published:** 2020-10-19

**Authors:** Severin Mühleder, Macarena Fernández-Chacón, Irene Garcia-Gonzalez, Rui Benedito

**Affiliations:** Molecular Genetics of Angiogenesis Group, Centro Nacional de Investigaciones Cardiovasculares (CNIC), Melchor Fernández Almagro 3, 28029 Madrid, Spain

**Keywords:** Endothelial cells, Senescence, Cell-cycle arrest, Sprouting, Vascular differentiation, Malformations

## Abstract

Therapeutic modulation of vascular cell proliferation and migration is essential for the effective inhibition of angiogenesis in cancer or its induction in cardiovascular disease. The general view is that an increase in vascular growth factor levels or mitogenic stimulation is beneficial for angiogenesis, since it leads to an increase in both endothelial proliferation and sprouting. However, several recent studies showed that an increase in mitogenic stimuli can also lead to the arrest of angiogenesis. This is due to the existence of intrinsic signaling feedback loops and cell cycle checkpoints that work in synchrony to maintain a balance between endothelial proliferation and sprouting. This balance is tightly and effectively regulated during tissue growth and is often deregulated or impaired in disease. Most therapeutic strategies used so far to promote vascular growth simply increase mitogenic stimuli, without taking into account its deleterious effects on this balance and on vascular cells. Here, we review the main findings on the mechanisms controlling physiological vascular sprouting, proliferation, and senescence and how those mechanisms are often deregulated in acquired or congenital cardiovascular disease leading to a diverse range of pathologies. We also discuss alternative approaches to increase the effectiveness of pro-angiogenic therapies in cardiovascular regenerative medicine.

## Introduction

In mammalian cells, highly complex and regulated processes involving a plethora of signals monitor the initiation, sustainment, and termination of cell division and migration. Due to its importance for tissue development, maintenance, and regeneration, several molecular mechanisms and checkpoints exist to regulate and balance these two morphogenetic processes. Through the regulation of cellular proliferation, the building blocks of a tissue are formed, while migration is essential to distribute those blocks in space, to give a tissue its ultimate shape and function. It is often difficult to dissociate the effect of genes or pathways on cell proliferation versus cell sprouting or migration in vivo. As a simple example, if the migration of cells towards a given growth factor niche is inhibited, proliferation will be indirectly also affected, since the cells will not be able to occupy areas of high growth factor bioavailability. On the other hand, if proliferation and formation of tissue building blocks are compromised, there will not be enough cells to move towards growth or chemotactic factors. A separate analysis of proliferation, sprouting, and migration is particularly necessary when studying the coordinated growth of blood vessels as certain cells of a growing vascular network fulfill one but not the other task. Most genetic studies so far simply assign a pro or anti-angiogenic function to a given gene or pathway. They suggest that genetic pathways either promote or inhibit both proliferation and sprouting of endothelial cells (ECs) [1–3]. This is either because most genes co-regulate in a similar fashion endothelial proliferation and sprouting, or they target mainly one of the two processes, but end up causing a similar deregulation of the other. However, several recent and higher resolution studies are proposing the existence of pathological situations or molecular mechanisms that when activated induce opposing proliferative and migratory cellular responses [4–7]. These can be mechanisms that when activated induce sprouting and cell migration at the same time as they block proliferation and vice versa. Besides a simplistic positive or negative role of specific molecular mechanisms on angiogenesis, it is also becoming clear that certain molecular mechanisms may have important roles only in some vascular development and disease contexts, and their effect is dose-dependent. Mechanisms that at low functional doses promote angiogenesis may be inhibitory at high doses and vice versa [6, 8]. Also, genes that can induce quiescence and vascular maturation when growth factor bioavailability is low or blood flow increases may be essential to sustain active angiogenesis when the vascular niche is high on growth factors and blood perfusion is lower. Vascular disease or aging, or the forced induction of angiogenesis and cell proliferation, may also induce replicative senescence, DNA-damage-induced senescence, or stress-induced senescence [9, 10].

Understanding how cell-type or cell-status-dependent gene function modifiers work in vivo and how they temporally intersect and modify a given genetic pathway function will be critical to overcome the failure of anti or pro-angiogenesis trials. It may also allow us to understand how mutations in certain genes lead to very different vascular malformations depending on the timing or vascular tissue in which they are identified [11–13]. Here, we will review some of the most basic and updated insights on the biology of ECs and discuss how those findings can be used to improve current therapies involving the inhibition or stimulation of blood vessel growth.

## Angiogenesis translational research

Angiogenesis research began with the first description of blood vessel growth in the late eighteenth century and received considerable attention in several observational studies later in the first half of the nineteenth century [14]. Approximately 75 years ago, experimental studies of mouse blood vessel growth during wound healing and tumor growth have sparked the idea that a secreted stimulating growth factor may be involved or even responsible for this process [15]. The authors furthermore hypothesized that tumor cells produce a putative factor of different quantity or quality than normal cells to accelerate vascular growth. Based on this theory, it was proposed that tumor and the corresponding blood vessel growth are interdependent processes, and targeting one mechanism would inevitably affect the other [16]. The cloning and biochemical characterization of a potent and specific human Vascular Permeability Factor or Vascular Endothelial Growth Factor-A (further named VEGF) then paved the way for the development of pharmacological compounds and therapies modulating angiogenesis with high specificity in a variety of human diseases [17]. The indispensable role of VEGF for vascular biology was later shown in multiple genetic studies conducted 25 years ago. These studies have shown that the deletion of a single *Vegf* gene allele or its main receptor (*Vegfr2*) caused the premature death of mouse embryos due to severe defects in vascular development [18–20]. Inhibition of VEGF signaling also significantly impaired vascular and tumor growth in mice [19, 21]. These results proved that the initial concept of VEGF being a key regulator of vascular tumor growth interdependence was correct. These revolutionary discoveries led to the development of several anti-angiogenic therapies targeting VEGF or its receptors, which prolonged the lives of numerous patients having tumors or prevented blindness due to age-related eye disease [22]. However, the clinical benefits of these treatments were below initial expectations and often due to anti-VEGF resistance [23–26]. It has since become apparent that the modulation of angiogenesis by VEGF (or anti-VEGF) is not as simple as initially thought, and that the physiological or pathological cellular context is a key modifier of VEGF function given the molecular cross-talk and compensation by multiple other signaling pathways. These complex molecular switches and balances need to be understood and integrated to stimulate or inhibit vascular growth in a clinically relevant manner.

Fortunately, since the first studies involving VEGF-targeted therapy in humans [27, 28], advances in research and diagnosis of physiological and tumor angiogenesis, vascular anomalies, and cardiovascular disease have shed light on other molecular mechanisms important for angiogenesis that could pave the way for the discovery of novel or more efficient therapeutic targets. These include the Notch [29–31], Ang-Tie, BMP/TGF-β [32, 33], EphrinB2-EphB4 [34, 35], Cxcr4-Cxcl12 [36], Wnt [37, 38], and various other factors involved in the endothelial metabolism [39–41]. These pathways are not as endothelial-specific as the VEGF pathway, but they control in one way or another endothelial proliferation, sprouting, or maturation. And like VEGF, their activation also often converges on the regulation of either the RAS viral oncogene homologue/mitogen-activated protein kinase (RAS/MAPK) or phosphoinositide 3-kinase/AKT/mammalian target of rapamycin (PI3K/AKT/mTOR) pathways [36, 42, 43]. It is interesting that the majority of the identified genetic mutations causing congenital or sporadic vascular malformations activate directly or indirectly either the RAS/MAPK or the PI3K/AKT/mTOR pathways [11]. These pathways are also highly relevant for cancer biology, and known anti-cancer drugs are being tested for their therapeutic potential against vascular anomalies [12, 44].

Even though inhibition of angiogenesis in tumors or endothelial overgrowth in vascular malformations has been so far an achievable target, the effective induction and promotion of angiogenesis have been so far much more challenging. This likely reflects the basic principle that is easier to bring down a house than to build one new. Understanding basic pro-angiogenesis principles will be crucial to achieve a clinical benefit for patients suffering from cardiovascular ischemic disease such as coronary artery disease or peripheral artery disease [45–48]. It will also be relevant for tissue regeneration, since poor blood vessel growth is one commonality in all types of chronic wounds leading to poor wound bed perfusion and granulation tissue formation [49]. Unfortunately, most therapies involving the use of vascular or endothelial mitogenic growth factors have failed in cardiovascular disease studies or did not reach the clinically relevant criteria of wound regeneration or closure [50, 51]. Therefore, understanding how to effectively induce maximal and sustained EC proliferation, sprouting, and vascular remodeling in desired tissues is of central importance to cardiovascular and regenerative medicine.

## Key regulators of endothelial sprouting

**Fig. 1:**
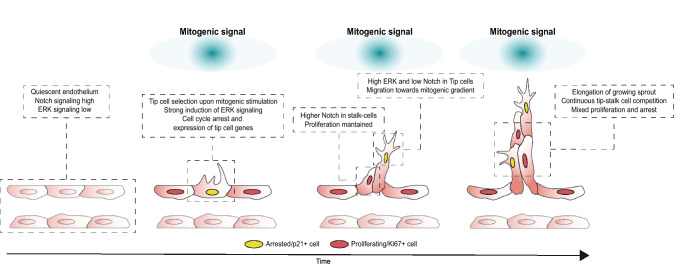
The role of cell cycle arrest during endothelial sprouting. Upon strong activation of quiescent ECs by a mitogenic signal, a tip cell becomes selected by induction of tip cell gene expression and temporary arrest of the cell cycle. Tip cells activate Notch in adjacent stalk cells which results in inhibition of their ERK activity, thereby maintaining their proliferative status. The arrested tip cells are responsible to guide the sprout towards the mitogenic gradient and are followed by proliferating cells that ensure the growth of the newly formed vessel

Tissues that grow beyond the diffusion limit of oxygen require an adequate blood supply to provide them with nutrients and oxygen and to remove waste products. The vascularization of such ischemic tissues occurs via a process termed angiogenesis, that consists in the formation of new blood vessels from existing ones [52, 53]. For angiogenesis to be effective, there must exist a balance between EC proliferation, sprouting, and migration, which involves a certain degree of endothelial differentiation or specialization (Fig. 1). Research in the last years led to the identification of morphological and molecular markers that distinguish endothelial tip cells from stalk cells and within these several subtypes. Tip cells are defined by their extended sprouting morphology and their position at the tip or leading edge of vessels [54, 55]. In central nervous system blood vessels (brain and retina), tip cells also emit long sensory filopodia likely for continuous evaluation of the surrounding microenvironment to adequately guide the sprout [56, 57]. However, the finding that blood vessel development occurs normally in a filopodia-deficient zebrafish model questioned the requirement of filopodia for tip cell guidance [58, 59]. Stalk cells, on the other hand, do not sprout, tend to proliferate significantly more than tip cells, and form the perfused network of capillaries at the angiogenic front [53].

Most of the insights on tip and stalk-cell biology were obtained in the postnatal retina angiogenesis model given its ease of genetic manipulation, dissection, immunostaining, and microscopic imaging [60]. The first studies in this system showed how sensitive tip cells and stalk cells were to the levels of VEGF signaling. VEGF is required for sprouting initiation, EC guidance, and also stalk-cell proliferation [55]. Signaling is primarily initiated via activation of the receptor VEGFR-2 [1, 53], although heterodimerization with VEGFR-3 has also been shown to be required for angiogenesis and sprouting [61]. Several different VEGF ligands binding to three different VEGF receptors exist [62]. This review will focus mainly on the role of VEGF-A-induced VEGFR-2 signaling which has been shown to be the most relevant ligand–receptor pair. For more information regarding the role of other VEGF ligands and receptors, the reader is directed to two recent reviews [22, 63]. Upon VEGFR-2 activation by VEGF, a series of phosphorylation cascades is triggered, including downstream MAPK (ERK) signaling activation [3, 63, 64]. Through yet unknown mechanisms, when the level of VEGFR-2 activation is very high, it results in the transcriptional upregulation of several genes that are enriched or highly expressed by endothelial tip cells. This includes the genes delta-like-ligand 4 (*Dll4*), *Esm1*, *Angpt2*, *Cxcr4*, *Apln*, *Kcne3*, *Igfbp3*, *Plxnd1*, *Robo4*, and *Adm* [65, 66]. Among these genes, the most tip-cell specific in angiogenic retina blood vessels is *Esm1*, likely due to the requirement for higher VEGF signaling levels for its transcriptional activation [3].

Several landmark studies have shown that through tip-to-stalk Dll4-to-Notch signaling, tip cells can further differentiate themselves from adjacent stalk cells [67, 68]. This mechanism may be important to amplify transcriptional and phenotypic differences among tip and stalk cells. Through this mechanism, cell-to-cell differentiation or specialization can occur even after a relatively small difference in VEGF signaling between tip and stalk cells. Even though all ECs express Dll4 at the angiogenic front, the tip-cell enriched ligand Dll4 induces a stronger activation of the receptors Notch (namely, Notch1 and Notch4) in adjacent stalk cells [31]. This results in the receptors cleavage and translocation of the Notch intracellular domain (NICD) to the nucleus where it binds the co-factor RBPJ (recombination signal-sequence binding protein Jk). This transcriptional complex elicits the expression of several direct Notch target genes, such as *Hey1* and *Hey2*, which are canonical transcriptional repressors that will directly or indirectly regulate the expression of hundreds to thousands of other genes [69–71]. This complex and multifactorial Notch-dependent transcriptional activation and repression mechanism is frequently associated with a change in a cell´s identity, fate, or proliferation [69, 71–73]. Its biological output is fast, variable, and often unpredictable, because it depends on the existent cell status and signaling context. In the case of ECs, the increase in Notch transcriptional activity is usually associated with a decrease in the cell´s activity and the adoption of stalk-cell features in the case of the angiogenic front, or the adoption of arterial cell features in the case of remodeling or mature vessels. This is confirmed by the fact that loss of *Dll4/Notch1/Rbpj* induces a significant increase in the number of sprouting cells (tip cells) and a loss of arterial identity [1, 6, 29, 31, 67, 68, 74–80].

Another important Notch ligand that regulates tip–stalk-cell differentiation is Jagged1. In angiogenic front ECs, this ligand behaves as a competitive and antagonistic Notch ligand [78]. This is due to the expression of Fringe glycosyltransferases (Mfng and Lfng) in angiogenic ECs. These enzymes glycosylate Notch receptors, turning their activation less sensitive to Jagged1 ligands and more sensitive to Dll4 ligands. The relatively higher expression of the stronger Dll4 ligand in tip cells and the weaker Jagged1 ligand in stalk cells reinforces the differences in the bidirectional Notch signaling between tip and stalk cells. In the absence of the weaker Jagged1 ligand, Dll4-Notch activity increases, and endothelial sprouting is suppressed [78].

Mechanistically, the suppression of endothelial sprouting by higher Dll4-Notch activity was initially thought to depend on the repression of VEGFR-2 transcription [31], a phenomena mostly observed in human umbilical vein ECs (HUVECs) under NOTCH overactivation [68, 81, 82]. However, several recent studies in zebrafish and mice have shown that physiological Notch signaling does not regulate *Vegfr2* transcription, translation, or phosphorylation in vivo [6, 77, 79, 83]. In contrast to *Vegfr2*, the transcription of the homologous *Vegfr3* receptor [80, 83] or its protein levels [77] were significantly upregulated after loss of Notch signaling in vivo, and thought to be sufficient to induce EC sprouting [61, 62]. However, later discoveries suggest that even though *Vegfr3* is an important gene for lymphatic sprouting [84], it may actually inhibit blood vessel EC sprouting [85–87]. Dll4-Notch activity has also been shown to regulate *Vegfr1* transcription [68], an essential modulator of embryonic vascular development [88]. VEGFR-1 kinase activity is relatively weak, but it has a significantly higher affinity for VEGF compared to VEGFR-2. Since it is secreted as a soluble form, it functions as a VEGF decoy receptor [1, 63, 89]. Indeed, mice expressing a mutated *Vegfr1* lacking its phosphorylation site develop normally [90], indicating that its core function may be to negatively balance VEGF signaling. Recently, several studies provided insights into the dynamics of VEGF signaling regulation by endothelial *Vegfr1* expression and its effects on morphogenesis and anastomosis formation, suggesting that it may function as a molecular rheostat [91–93].

Despite the controversy surrounding the mechanistic cross-talk between Notch and VEGFR signaling, it is clear from several recent studies conducted in zebrafish and mice that Notch suppresses the downstream MAPK/ERK signaling [6, 64], by yet unidentified mechanisms and independently of decreases in Vegfr2 signaling [6]. This observation is in line with the fact that stalk cells have significantly more Notch and less ERK activity than tip cells. Since the differences in ERK signaling between tip and stalk cells are far more pronounced than the observed differences in Vegfr2 or Vegfr3 mRNA or protein levels, the expressions of these genes are likely not polarizing or key differentiation mechanisms. Availability and distribution of the VEGFA ligand and *Nrp1* expression, another known modulator of VEGF signaling, seem to have a much higher tip–stalk differentiation effect [55, 94, 95]. Tgf-beta/Bmp/Alk signaling and its mechanistic interaction with Notch and Nrp1 also seem to be highly relevant for tip–stalk-cell differentiation [94]. All these studies, however, have not clearly addressed the paradoxical evidence, showing that stalk cells have significantly lower VEGF/ERK activity, even though they proliferate more than tip cells [6, 55].

## Bell-shaped response to angiogenesis stimulation and its implications

The most prominent pathway shown to regulate EC proliferation is VEGF signaling via VEGFR-2 [17, 22, 63, 96]. Similar to many other growth factor signals, VEGF signaling via VEGFR-2 leads to the downstream activation of the MAPK pathway and phosphorylation of ERK1/2 which is widely regarded as a pro-mitogenic pathway [63]. However, ERK1/2 phosphorylation is significantly higher in tip cells [6, 64] and these cells are significantly less proliferative than stalk cells [55], particularly the ERK/Esm1-high tip cells [6]. Furthermore, in vitro analyses revealed that endothelial motility, a key feature of endothelial tip cells [31], is enhanced when ECs are in the G0/G1 phases of their cell cycle and decreased in their proliferative S/G2/M phases [97]. It has also been shown in vivo that the expression of a constitutively active mutant of *Vegfr2* in stalk cells induces extreme tip-cell features, such as very high ERK activation and a pronounced cell-cycle arrest [6]. Interestingly, inhibition of Notch signaling was shown recently to have temporal and context-dependent effects on EC proliferation that are dependent on the heterogeneity of ERK signaling levels [6]. When Notch signaling is blocked in highly proliferative ECs located at the angiogenic front, these have an increase in ERK signaling to levels similar to tip cells, and this is detrimental for their proliferation, because it leads to the strong upregulation of the cell cycle inhibitor p21 (encoded by *Cdkn1a*). However, when Notch signaling is blocked in VEGF low and quiescent retina vessels, these have a milder increase in ERK levels, which induces cell cycle entry. This work shows that there is a bell-shaped response to ERK/mitogenic stimulation and explains why tip cells proliferate significantly less than adjacent stalk cells, even though they have more VEGF/ERK signaling [6]. The identified ERK-dependent hypermitogenic cell cycle arrest mechanism is highly conserved and also occurs in other cell types and in cancer [98].

These data imply that the effect of VEGF or any other pro-mitogenic stimulus that induces high MAPK/ERK activity, such as inhibition of Notch signaling, may not lead to the intended result of increasing angiogenesis. In fact, Notch inhibition [6], or VEGF ocular injection [99], has been shown to induce transient vascular sprouting and expansion, in the absence of proliferation. If sustained over time, excessive mitogenic stimulation leads to a pronounced decrease in vascular outgrowth and angiogenesis, since the vessels lose the capacity to maintain their proliferation in VEGF-rich and hypoxic areas [6]

This bell-shaped dose–response to mitogenic stimulation has not been noticed in most previous studies because of the focus on markers of cell proliferation and not on markers of cell-cycle arrest. VEGF administration to a quiescent vascular network should always cause an increase in the frequency of proliferating (KI67+/EdU+) ECs, because the baseline level of EC proliferation is very low. However, given the recently identified EC hypermitogenic arrest marker p21 [6], it will be of interest to analyze how prevalent is the cell-cycle arrest in tumor vessels or in situations of therapeutic pro-angiogenesis. Could the hypermitogenic arrest explain the failure of most pro-angiogenesis therapies so far [47, 48, 98, 100, 101]?

Exploiting pro-mitogenic stimulation for therapeutic angiogenesis is a promising strategy for treating patients suffering from ischemic diseases such as cardiovascular disease or chronic wounds. This would promote the induction of tissue vascularization and thus better tissue function or regeneration [102]. Cardiovascular disease remains a leading cause of death or morbidity worldwide [103] and every year millions of patients are suffering from wounds that are the result of traumatic injury or surgical routine in the US and Europe [52]. Effective treatment options and understanding the cardiac or wound healing process are therefore of central clinical concern. Ineffective angiogenesis can lead to impaired cardiac and wound healing as several regenerative and tissue homeostasis processes are affected in the absence of the vascular function [47–49, 100, 101, 104, 105]

In regenerative medicine, VEGF delivery has been tested numerous times with the goal of achieving increased tissue vascularization and regeneration, however, without reaching any clinically relevant outcome [47–49, 100, 101, 104, 106, 107]. One of the hypotheses suggests that rapid diffusion and short half-life of VEGF in vivo may explain the absence of significant benefits in humans [108]. In other cases, applications of high doses or multiple growth factors lead to the formation of unstable, tumor-like vasculature and leaky vessels [109]. As described above, VEGF and Notch-driven angiogenesis is a tightly balanced and coordinated process. Mouse genetic experiments have shown that even a 50% decrease in *Vegf* or *Dll4* expression and signaling leads to severe vascular defects and embryonic lethality [18, 19, 75, 76, 110]. Indeed, the microenvironmental VEGF concentration has been shown to determine the threshold between normal and pathological angiogenesis [111]. Several studies have already pointed to the importance of a balanced VEGF and Notch signaling dose for therapeutic angiogenesis, and suggested that intermediate or lower mitogenic stimuli may be more effective than hypermitogenic stimulation [47, 54, 101, 111, 112]. Delivery of high local VEGF concentrations led to angioma-like vessel formation, while lower doses caused functional vessel formation [47, 111, 113]. This may explain why the use of VEGF in therapeutic angiogenesis in patients remains to be experimental and is not a standard-of-care treatment [47]. For example, the current clinical procedures for wound management involve surgery, removal, and debridement of necrotic tissue and biofilms and application of wound dressings to promote endogenous wound repair [114]. Instead of VEGF, only one mitogenic growth factor was approved in 1997 to be used in a gel for treating chronic wounds: platelet-derived growth factor (PDGF)-BB and it still remains to be the only pharmacological agent to be used today for this indication [114, 115]. In addition to wound healing, patients suffering from chronic cardiovascular diseases such as coronary artery disease, peripheral artery disease, or refractory angina could benefit from pro-angiogenic therapies to promote tissue regeneration by collateral vessel formation [47, 107]. However, several clinical trials employing infusion or gene transfer of VEGF failed to show any clinically relevant benefit for these patients [47, 48, 101, 106]. This body of data collectively suggests that balancing mitogenic stimuli dose and its downstream effectors or modifiers will be critical to enhance EC proliferation and thus functional angiogenesis. However, this balance that is beautifully orchestrated by developing tissues may be difficult to achieve in therapeutic settings of angiogenesis, where we can only add pro-mitogenic pharmacological compounds, without knowing or being able to monitor how they are actually modulating EC proliferation throughout the treatment. Particularly problematic may be that the occurrence of an endothelial hypermitogenic cell cycle arrest is time-, context-, and tissue-dependent and thus difficult to control for. It also has remained unnoticed in most previous studies because of the focus on markers of cell proliferation and not on markers of cell-cycle arrest as mentioned above. In most cases, an intense and transient stimulation with an endothelial mitogen such as VEGF results in a strong ERK activation and induction of EC proliferation, however, often to a level that cannot be sustained leading to a decrease in cycling ECs over time, as it has been shown in different in vivo models [6, 113]. Furthermore, mitogenic stimulation does not affect all areas of a growing vascular tree equally, since mitogen-induced cell cycle arrest has been observed primarily at the angiogenic front, while an increase in proliferating ECs can be observed in more mature and quiescent vascular regions [6]. Finally, high mitogenic stimuli may be productive and induce vascular network growth through different processes. For instance, VEGF overexpression in skeletal muscle cells can result in vessel formation through splitting angiogenesis or intussusception, a process that occurs without the formation of endothelial tip cells [116].

The concept of balanced angiogenesis also clashes with the ideal and simplistic concept of achieving maximal EC proliferation and angiogenesis *à la carte*. One alternative to the difficult goal of achieving a balance in mitogenic stimulation is to simultaneously induce high mitogenic stimulation and inhibit the endogenous hypermitogenic arrest mechanisms, such as the ones mediated by classical cell cycle inhibitors [98], or by exploiting alternative pathways that limit endothelial proliferation [113, 117]. These strategies may allow a significant boost in pro-angiogenesis therapies. But before it is important to understand what are the main players in the hypermitogenic arrest, what pharmacological tools we have to target them, and if their targeting has any negative consequences for the endothelial health and genomic stability.

## Role and disease relevance of canonical cell cycle inhibitors

**Fig. 2:**
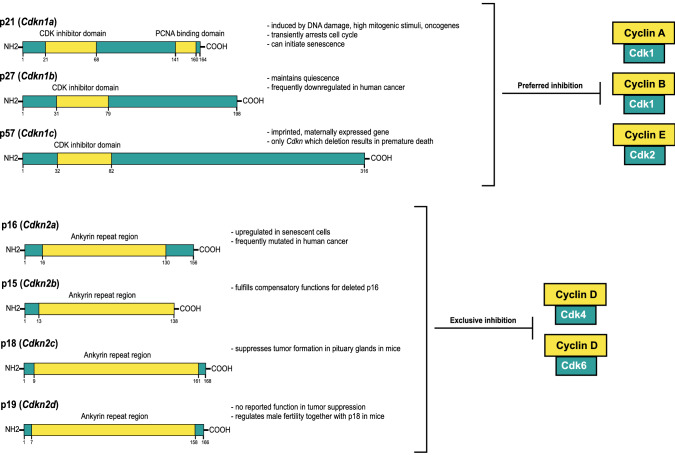
Structural domains of Cdkn proteins. Members of the *Cdkn1* family have a specific Cdk inhibitor domain region. This domain is of similar size in all three Cdkn1 proteins. p21 additionally contains a PCNA-binding domain. All members of the *Cdkn2* family contain an ankyrin repeat region used for binding to and regulating the function of binding partners such as Cdk. This region is of similar size in p16 and p15 and in p18 and p19. Cdkn2 proteins exclusively inhibit the complex formation of D-type cyclins with Cdk4/6. Members of the *Cdkn1* gene family can inhibit all cyclin/Cdk complexes but primarily block the activity of Cdk1 and Cdk2

Several factors exist which control the status and progression of a cell cycle as dysregulated cell proliferation can be harmful to cells or leads to cancer [44]. In mammalian cells, this is governed by three classes of molecules, cyclin-dependent kinases (CDKs), cyclins, which serve as activators of CDKs to exert their kinase activity, and CDK inhibitors [118]. While only one major CDK appears to be sufficient for cell division of yeast, more than 20 CDKs and several different cyclins exist in mammalian cells that can form various combinations of complexes and are expressed differently in each phase of the cell cycle [118, 119]. With the advent of gene knockout mouse technologies, it became evident that a great degree of redundancy exists within this network of cell cycle proteins [118]. Whenever the conditions are unfavourable or risky for cell division, CDK activity and thus cell cycle progression can be restrained by several canonical cell cycle suppressors, named as cyclin-dependent kinase inhibitors (*Cdkn*) that will be the focus of this review. Most of what we know today about the function of these genes has been studied in the context of cancer and in vitro. However, *Cdkn* genes also have important physiological and tissue homeostasis roles. The first findings in vitro were followed by the characterization of mouse lines with a single or multiple *Cdkn* genes deleted, which enabled the evaluation of their physiological roles in vivo and the identification of genetic compensation among some of the homologous Cdkn proteins [44, 120–122]. *Cdkn* genes can be divided into two separate families: genes which belong to the *Cdkn1* or Cip/Kip family encode the cell cycle regulators p21 (*Cdkn1a*), p27 (C*dkn1b*), and p57 (*Cdkn1c*) [123]. On the other hand, genes of the *Cdkn2* or INK4 family encode the proteins p16 and p19ARF (*Cdkn2a*), p15 (*Cdkn2b*), p18 (*Cdkn2c*), and p19 (*Cdkn2d*) [123, 124]. Due to an alternative reading frame of the *Cdkn2a* gene, an unrelated gene *ARF* is encoded expressing a protein named as p14ARF in humans and p19ARF in mice [125], which shares the ability to induce cell cycle arrest with other members of the *Cdkn* families [126]. Despite their similar names, *Cdkn1* and *Cdkn2* genes code for biochemically and functionally distinct proteins [127, 128] (Fig. 2). CDKN2 proteins specifically inhibit CDK4 and CDK6 kinase activity, while CDKN1 proteins preferentially inhibit CDK1 and CDK2 but can block the activity of all cyclin/CDK complexes [44, 118, 119].

Global knockout experiments in mice confirmed the role of some of these *Cdkn* genes in suppressing tumor growth and gave insights into their tissue functions [124, 127]. Due to their role in the expansion of cell lines in vitro and their expression and regulation patterns in vivo, it was assumed that factors from the *Cdkn1* and *Cdkn2* families would have an essential function during the normal development of tissues in addition to their role in cancer [127]. However, p21 (*Cdkn1a*) [129], p27 (*Cdkn1b*) [130–132], p16 (*Cdkn2a*) [133, 134], p15 (*Cdkn2b*) [135], p19ARF (*ARF*) [136], p18 (*Cdkn2c*) [135], and p19 (*Cdkn2d*) [137] global KO mice develop normally, which raised questions about their in vivo physiologic role [123]. In contrast to these, only 8.5% of *Cdkn1c* KO mice reach adulthood due to cleft palate, gastrointestinal, and endochondral ossification abnormalities [138, 139]. However, additional information on *Cdkn* gene function could have been masked due to the global knockout technology, but might be revealed with genetic mosaics [5]. For example, a global deletion of *Cdkn1b* has recently been demonstrated to result in only a 0.6-fold increase in corneal EC proliferation in vivo, while the simultaneous presence of wild-type and mutant clones within one tissue revealed a sixfold expansion of mutant cells compared to wild-type cells [140].

Although little is known about the significance of *Cdkn* genes in cardiovascular biology, most studies suggest a role for *Cdkn1* and *Cdkn2* genes in suppressing cell proliferation, maintaining tissue homeostasis, and genetic stability [141, 142]. A prominent role of the tumor suppressor p53 in guarding the stability of the genome by inducing expression of *Cdkn* genes has been described in numerous studies (reviewed here [143]). However, certain *Cdkn* genes such as *Cdkn1a*, *Cdkn1b*, *Cdkn2a*, *Cdkn2b*, and *ARF* have also important p53-independent functions in ensuring cell cycle arrest and DNA repair mechanisms [124, 144–146].

Genetic deletion of *Cdkn1a* or *Cdkn1b* does not result in major developmental or proliferation defects but results in spontaneous tumor formation in some tissues of adult mice [120]. *Cdkn1a* and *Cdkn1b* double KO mice also develop normally and reach adulthood, but have a decreased survival due to an increased tumorigenesis rate, suggesting some degree of compensation or functional overlap between these *Cdkn* genes [120, 147]. Indeed, both genes have been observed to be significantly downregulated within cancer cells [148, 149]. As mentioned above, *Cdkn1c* deletion results in severe defects in cleft palate, gastrointestinal, and bone development [138, 139]. Because of this, compensation between all three *Cdkn1* genes has mostly been observed at earlier stages of development. Deletion of *Cdkn1a* in addition to *Cdkn1c* results in a twofold increase in lethality at E16.5 due to defects in skeletal muscle cell proliferation and differentiation [150]. Similarly, combined deletion of *Cdkn1b* and *Cdkn1c* has also been reported to result in a twofold increase in lethality between E12.5 and E16.5 compared to *Cdkn1c* single deletion, although the only phenotypic difference was observed in the development of the eye lens [150]. Furthermore, this wide developmental window and incomplete phenotypic penetrance is likely the result of genetic-background-dependent modifiers [151] which makes these data difficult to compare to other single *Cdkn* knockout studies. Interestingly, when analyzing *Cdkn1* single, double- and triple-gene global KO mice in a single study, it was suggested that *Cdkn1a* only has an accessory role, while the compensatory functions of *Cdkn1b* appear to be more critical during the development of *Cdkn1c* knockout mice [152]. Specifically, a triple *Cdkn1* KO results in 100% lethality, while the double KO of *Cdkn1a/c* or *Cdkn1b/c* leads to 19% and 67% lethality, respectively, at E15.5. These compound mutant embryos show a significant increase in the proliferation and apoptosis of different cell types. The simultaneous increase in proliferation and apoptosis may explain why the tissues of these animals show no apparent signs of overgrowth.

Analysis of mice with deletion of individual *Cdkn2* genes revealed that alone they have no major functions in tissue development, likely due to genetic redundancy or compensation. However, in contrast to *Cdkn1* genes, *Cdkn2a/b* is one of the most frequently mutated gene loci in human cancer [141], suggesting a crucial function of these genes in maintaining genomic stability of quiescent cells. Single deletion of *Cdkn2b* (codes for p15Ink4b) in mice does not result in any significant tumor susceptibility [135], while *Cdkn2a* (codes for p16Ink4a and p19ARF) global knockout animals reach adulthood without any major developmental defects, but begin to spontaneously form tumors at 20 weeks of age [153]. In line with this, experiments in mice showed that an increased gene dosage of the *Cdkn2a* locus provides the animals with resistance to cancer while maintaining the same life span [154]. Interestingly, it appears that Cdkn2b fulfills a compensatory function in the absence of p16 and vice versa, while the deletion of *p19ARF* does not seem to be compensated for [135, 155]. This could explain the reduced tumor-free survival of *p19ARF*-null compared to *p16Ink4a*-null mice [156] and highlights the independent function of p19ARF [144].

Data on *Cdkn2c* or *Cdkn2d* gene KO mice indicate no function on tissue development and only little contribution to adult cells homeostasis and genomic stability. *Cdkn2c* knockout mice develop normally and age well into adulthood, but have an increased body size and hyperplastic spleen and thymus and develop pituitary tumors by 10 months of age [157]; however, its function is partially compensated by the *Cdkn2a* gene [158]. No tumor phenotypes have been observed in *Cdkn2d* knockout animals [137].

Besides data on single *Cdkn2 gene* deletions described above, studies in mice with multiple *Cdkn2* genetic deletions revealed compensatory or redundant roles of certain *Cdkn2* genes. In contrast, the deletion of *Cdkn2b* in addition to *Cdkn2c* does not lead to significantly different phenotypes in mice indicating limited compensatory tasks of these two *Cdkn* genes [135]. Importantly, multiple studies suggested that co-deletions of *Cdkn2c* and *Cdkn2a/b/ARF* observed in some human cancers such as glioblastoma are responsible for worsened tumor severity (reviewed in [159]). To our knowledge, the effects on development or tumor formation of a combined deletion of all four *Cdkn2* genes has not been investigated so far.

Despite the structural differences, the above-described mild phenotypes observed upon global KO of single or few Cdkn1 (Cip/Kip) or Cdkn2 (Ink4) proteins suggest possible compensatory mechanisms between *Cdkn1* and *Cdkn2* genes. The functions of both Cip/Kip and Ink4 cell cycle regulators converge at the regulation of Cdk4 and Cdk6, which can be inhibited by any cell cycle inhibitor of the *Cdkn* gene family [44]. In fact, p16 and p21 compete for Cdk4 binding [160], suggesting that removal from one binding partner could shift the equilibrium towards other available binding partners [161] potentially leading to confounding effects. Therefore, to reveal masked compensatory pathways between Cip/Kip and Ink4 proteins, multiple genetic deletions or mutation studies are needed to better understand the complex network of cell cycle inhibition.

In the particular case of the cardiovascular system, studies in zebrafish embryos and mice have uncovered specific roles for *Cdkn* genes in angiogenesis [6, 162, 163], vascular homeostasis [120, 164], or atherosclerosis and aging [165–167]. scRNA-seq data revealed expression of *Cdkn1* and *Cdkn2c/d* genes in quiescent ECs, pericytes, and SMCs [168, 169]. Interestingly, *Cdkn2a/b* genes, required for the genomic stability of cells [142, 154, 155] and markers of geriatric cellular aging and senescence [10, 124] are not expressed in quiescent vascular cells of young mice [168, 169]. On the other hand, expression of *Cdkn1a/b* genes varies greatly in single capillary, venous, and arterial mouse lung ECs, interestingly, in a similar fashion as proliferating cells expressing *Pcna* [168, 169]. However, it is necessary to mention that *Cdkn1* genes are subject to tight post-translational regulation [170], and hence, their transcription dynamics might not accurately reflect on the presence of the protein or its function.

**Fig. 3:**
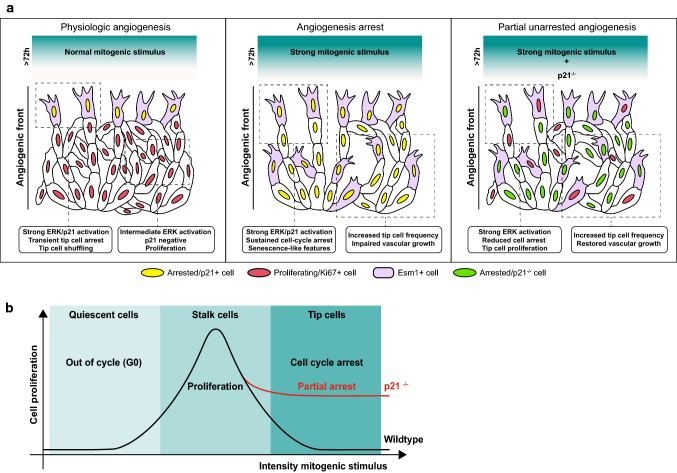
The role of mitogenic stimuli and p21 in endothelial cell cycle arrest. **a** At the angiogenic front, p21+ tip cells arrest and are followed by proliferating Ki67+ stalk cells. If the levels of the mitogenic stimulus are high in all cells, the phenotypic differences between tip and stalk cells disappear as most cells at the angiogenic front become arrested and acquire tip-like features. This cell-cycle arrest at the angiogenic front impairs the sustained vascular network growth despite an increased number of tip cells. If p21 function is lost or inhibited, a high mitogenic stimulus fails to induce cell-cycle arrest in some cells, resulting in the partial preservation of proliferation at the angiogenic front. **b** The relation of EC proliferation to the intensity of a mitogenic stimulus corresponds to a bell-shaped curve. Depending on the intensity of mitogenic stimulus, cells can be either quiescent, proliferate, or become arrested. EC proliferation is maximal at intermediate mitogenic stimulus. Proliferating ECs become arrested if a threshold is exceeded which triggers the upregulation of p21 and inhibition of proliferation. This hypermitogenic cell cycle arrest can be partially prevented by p21 deletion in ECs, resulting in proliferative activity even at high mitogenic stimulus levels

Data from EC culture experiments initially suggested that p21 is a positive regulator of proliferation and downregulated in a Notch-dependent manner as these cells reach confluency [163]. This puzzling observation contrasts with what was shown in keratinocyte, fibroblasts, and tumor cell cultures [145, 171–173]. More recently, it was confirmed that p21 does not have any major function on EC proliferation during developmental angiogenesis in vivo, except in endothelial tip cells, or after the stimulation of angiogenesis by increasing VEGF signaling or decreasing Notch signaling [6]. This is because during vascular development, the p21 protein (unlike the mRNA) is not normally expressed by the large majority of ECs, except by endothelial tip cells [6], which undergo hypermitogenic arrest induced by excessive VEGF/ERK stimuli. It was additionally identified that the VEGF-mediated p21 induction induces endothelial sprouting, but ultimately halts angiogenesis due to the inhibition of endothelial proliferation. This cell cycle arrest mechanism is restricted to tip ECs during physiological angiogenesis. Accordingly, in the absence of p21, tip ECs proliferate significantly more, whereas ECs with a balanced mitogenic stimuli (stalk ECs) proliferate normally, since they do not express p21 protein. These results suggest that pro-angiogenic therapies employing delivery of VEGF may be counterproductive, as it can induce p21 and cell cycle arrest of angiogenic ECs closest to hypoxic areas, which are the most important cells for effective tissue vascularization (Fig. 3a). However, targeting the function of p21 or other cell-cycle checkpoints at the same time VEGF is provided or Notch is inhibited, may lead to more effective EC proliferation and angiogenesis in wound healing or after myocardial infarction/ischemia (Fig. 3b). The general role of p21 in tissue regeneration appears to be ambiguous as studies exist which report an improved [174], impaired [175], or indifferent [176] regenerative potential upon *Cdkn1a* deletion. Furthermore, data from global heterozygous *Cdkn1a*^+/−^ and homozygous *Cdkn1a*^−/−^ animals indicate that both partial and full deletion of *Cdkn1a* improves neovascularization in a subcutaneous disc angiogenesis system [177]. In contrast to angiogenic tip cells, p21 protein is undetectable in more mature quiescent vessels [178]. However, its low basal or very sporadic and transient high expression is still important to maintain long-term vascular quiescence, since approximately 9% of *Cdkn1a* KO mice have been reported to develop hemangiomas at an average age of 16 months [164].

In addition to p21, p27 is also capable of inhibiting all complexes of cyclin with CDK, thereby preventing abnormal proliferation [179]. Because the regulation of p27 expression is often dysfunctional in human cancer, its expression levels in tissues were proposed as a prognostic marker after chemotherapy [180]. Yet, a role for the cell cycle regulator p27 in angiogenesis remains enigmatic. Due to the highly similar structural homology between p21 and p27, they may compensate for one another. Indeed, p21 and p27 have comparable cell cycle mechanisms of action [179, 181]. However, data from tumor suppression experiments in global knockout mice suggest that different upstream regulation mechanisms exist for p21 and p27 [182]. Additionally, it was shown in vivo that p21 activity in hepatocytes can compensate for the absence of p27 [183]; however, this appears to be in conflict with data from other in vivo studies, showing that p27 can only partly [184] or not at all be compensated by p21 [185]. A recent study showed that shear stress-dependent activation of Notch in ECs resulted in the upregulation of p27 which induced EC quiescence and arterial specification in vivo [186], even though *Cdkn1b* KO mice develop arteries [130–132]. Interestingly, inhibition of p27 in developing zebrafish embryos resulted in an increased number of ECs in developing vessels, however, without impairing the formation of intersegmental blood vessels [162]. Nevertheless, a role for p27 in the growth arrest of angiogenic tip ECs experiencing high VEGF levels was never shown, but cannot be ruled out.

Like p21 and p27, p57 also belongs to the Cip/Kip family thus sharing similar features (Fig. 2) [127]. However, p57 is the only *Cdkn* gene to be required for skeletal muscle, cleft palate, and gastrointestinal tissue development, resulting in severe embryonic defects upon deletion and death shortly after birth [138, 139]. As a result of this, there are very limited data on its role in neonatal or adult tissues. Interestingly, using tissue-specific knockout mice, a cooperation between *Cdkn1a–Cdkn1c* and *Cdkn1b–Cdkn1c* in hematopoietic stem cell quiescence has been reported, suggesting that *Cdkn1* genes can indeed compensate for each other if co-expressed in the same cell [187, 188]. Given their broad co-expression in different cell types composing the vascular tissue, it will be important to determine the combinatorial roles of *Cdkn1a/b/c* genes in distinct vascular cells during cardiovascular development, homeostasis, and disease.

Like *Cdkn* genes, the tumor suppressor p53 can also induce cell cycle arrest, prevents apoptosis, and promotes DNA repair mechanisms. p53 is considered an upstream regulator of other cell cycle regulators such as p21 [189]. In heart disease, p53 has been suggested to be a central mediator of angiogenesis, and upregulation of this factor may contribute to the rarefaction of the cardiac vasculature which was observed in hypertrophic hearts (reviewed in [190]). Of note, it has been demonstrated in various models that endothelial-specific deletion of p53 in mice resulted in increased vessel formation not during tissue development, but in hypertrophic hearts, resulting in improved cardiac function and prevention of heart failure [191]. The same study also demonstrated that deletion of p53 augmented angiogenesis during hindlimb ischemia. These results suggest that p53 can restrict EC proliferation and that blocking p53 could promote angiogenesis in cardiovascular disease. In addition to vessels, p53 has been shown to be transiently activated in cardiac tissue during regeneration in neonatal mouse hearts [192]. A recent article has shown that knockdown of protein tyrosine kinase phosphatase-1B (PTP1B) promotes EC senescence by upregulation of p53 and p16 [193]. This is particularly interesting, since PTP1B has also been shown to inhibit VEGF-induced VEGFR-2 and ERK1/2 phosphorylation [194]. Taken together, this indicates that dysregulation of this phosphatase drives EC senescence through aberrant activation of VEGFR-2 and ERK1/2. On the other hand, it has been shown that inactivating genetic mutations in the endothelial phosphatase *PTPRB*, another negative regulator of VEGFR-2 signaling, occur in about 26% of malign vascular tumors, named angiosarcomas [195]. This ambiguity of the VEGFR-2 signaling pathway in inducing endothelial proliferation and senescence may be related to the bell-shaped response to mitogenic stimulation discussed above [6].

Little is known about the contribution of the cell cycle regulators *Cdkn2a* (p16Ink4a and p19ARF) *Cdkn2b* (p15Ink4b), *Cdkn2c* (p18Ink4c), and *Cdkn2d* (p19Ink4d) to endothelial sprouting, proliferation, or hypermitogenic arrest. Although one of the most common genetic alterations in human cancer is the deletion of the *Cdkn2a/b* gene locus encoding for the cell cycle regulators p15Ink4b, p16Ink4a and ARF-Ink4a [44, 141], it seems that these factors do not play a major role in EC development and physiology. In muscle stem cells, p16 was also shown to be upregulated in geriatric mice and a key factor in the loss of regenerative potential [196, 197]. Interestingly, while no developmental vascular defects occur upon *Cdkn2a* deletion in mice, 23% of all spontaneously formed tumors in these animals were angiosarcomas [156], suggesting a critical function of this *Cdkn* gene in maintaining EC homeostasis. Importantly, this was not described upon deletion of *ARF* or the entire *Cdkn2a/ARF* gene locus [156]. Indeed, another report showed that deletion of *Cdkn2a/ARF* led to hemangioma formation in only 6.1% of all tumors analyzed [198], suggesting that co-deletion of *ARF* changes the spectrum of tumors caused by *Cdkn2a* deletion in addition to an increase in lethality [156]. A recent study analyzed tumors from animals overexpressing mutated Cdk4 or Cdk6, which severely impairs the function of all four *Cdkn2* cell cycle regulators but not of p19ARF [199, 200], and found that 56% of all tumors in these animals were angiosarcomas [122, 161]. Together, these data indicate a link between *Cdkn2* genes and the long-term maintenance of EC quiescence.

Still, future combinatorial and more refined loss-of-function studies of *Cdkn2* genes may reveal the existence of genetic redundancy and even more important physiological roles in cardiovascular development, homeostasis, disease, or aging. Despite this body of data describing the importance of *Cdkn* genes in cellular processes, including vascular growth and homeostasis, it is still not possible to directly and selectively target Cdkn proteins such as p21 with existing pharmacological compounds [201]. Therefore, it will be also of high relevance to use these as biomarkers for the discovery of alternative or other upstream/downstream molecular mechanisms leading to the hypermitogenic arrest or senescence of ECs. These novel molecular mechanisms may be easier to target or have a more vascular or hyperangiogenesis-specific function, reducing potential side-effects and increasing the value and specificity of pro-angiogenesis therapies. In other circumstances, such as during tumor angiogenesis, it may be also of high therapeutic relevance to modulate these mechanisms to induce irreversible EC arrest or senescence, as an alternative to the current induction of endothelial quiescence with standard anti-angiogenesis (anti-VEGF) compounds. Endothelial senescence by definition is not reversible, and its induction may be able to generate long-lasting effects, unlike the reversible induction of endothelial quiescence by current anti-angiogenesis therapies. In addition, it is known that ECs can use alternative sources of stimulation when VEGF or other sources are blocked [41], but if they have activated a cell cycle arrest or pro-senescence mechanism, this may provide an insurmountable break for angiogenesis.

## Endothelial senescence and aging in cardiovascular disease

Cellular senescence is characterized by an irreversible cell cycle arrest along with metabolic changes and alterations of a cells’ secretome induced by a variety of stressors such as DNA damage or replicative stress. Senescence is different from quiescence as senescent cells remain unable to respond to any mitogenic stimuli and are therefore incapable of re-entering the cell cycle [202]. It is generally believed that during aging, there is a continuous increase in the number of senescent cells throughout all tissues of the body resulting in a progressive decrease of an organ proliferative capacity or function. Senescence impairs a tissues’ resilience to disease and ability to regenerate after injury [203]. However, in some contexts, senescent cells have also been reported to have important developmental, homeostatic, or health-promoting functions [176, 204–206].

In cancer biology, various types of senescence have been identified: replicative senescence, DNA-damage-induced senescence, stress-induced senescence, and oncogene-induced senescence [202, 204, 206]. This may indicate diversity in the underlying molecular mechanisms, which may also vary depending on the cell type and environmental conditions. For example, overexpression of the micro RNA 21 (miR-21) has been observed several human tumor types including brain, breast, and cervical cancer [207], but its enhanced expression in ECs induces cell cycle arrest by increasing p21 [208]. p21 is one of the most important markers of cell cycle arrest or cellular senescence, and it is frequently downregulated in cancer [145, 209]. However, its reliability as a bona-fide senescence marker remains controversial. It has been demonstrated that short-term expression of p21 leads to a reversible growth arrest, while only a sustained and high expression of p21 induces irreversible cell cycle exit [171]. Expression of p21 appears to be important for the initiation of senescence; however, it is not maintained in some senescent cells unlike p16, which seems to accumulate at high levels only in senescent cells [210], although a certain dynamic expression of this factor has been described as well [211]. The senescence mechanisms and markers may also vary according to the cell type and cellular context. Besides the expression of these two genes, other unique features of senescent cells are the very active biosynthetic activity and the expression of a variety of cytokines (senescence-associated secretory phenotype), larger cell size, high beta-galactosidase activity, heterochromatin formation, and increased reactive oxygen species (ROS) levels [204]. Importantly, constitutive, irreversible activation of the MAPK signaling pathway in fibroblasts appears to be essential for executing a senescence program in vitro [212–214]. In ECs, senescence leads to the development of a dysfunctional phenotype, which promotes impaired tissue function by acquiring pro-oxidant, pro-inflammatory, and pro-thrombotic features in addition to irregular blood pressure regulation and cell cycle arrest [203, 215]. An accumulation of senescent cells has been regarded to be a significant contributor to cardiovascular disease in an aging population [215]. The expression of the cell cycle suppressors p53 and p21 in arteries of healthy individuals correlates with age [216, 217] and is even more increased in vessels from hypertensive patients [218], suggesting a link between cell cycle arrest (senescence), aging, and cardiovascular disease. Additionally, it has been claimed that p16 levels are increased in aged human veins and aged mouse aorta tissue [216, 219]. Importantly, an increased expression of p53 has been associated with several age-related conditions such as heart failure, atherosclerosis, obesity, and diabetes (reviewed in [215]). Indeed, global expression of a mutated, constitutively active form of p53 in mice resulted in a significantly decreased tumor incidence but decreased survival due to early onset of age-related defects such as impaired tissue regeneration, general organ atrophy, osteoporosis, and reduced stress tolerance [220]. Moreover, endothelial-specific deletion of p53 in mice has been shown to have no impact on the normal development of vessels, but improves cardiac neovascularization and regeneration after heart failure [191]. In another study, a global increased gene dosage of p53 in mice, resulting in normally regulated basal but increased stress-response levels, did not result in increased prevalence of age-related diseases but in normal aging and enhanced resistance to chemically-induced fibrosarcomas, papillomas, and urinary bladder carcinomas [221, 222]. Similarly, in a mouse cardiac regeneration model, significantly elevated p53 levels were detected in heart tissue of young animals, while in adults only, a mild induction was observed [192]. Collectively, these data suggest that both the intensity (strong or weak) and the dynamics (transient or sustained) of p53 induction may be relevant to promote age-related defects. Interestingly, in mice containing an increased gene dosage of *Tp53*, p21 levels were also increased compared to WT animals upon induction of DNA damage [222], pointing out the link between p53 and p21 [223, 224]. Using a similar approach, mice containing an increased gene dosage of *Cdkn1a* display an indistinguishable cancer protection phenotype to mice containing an additional allele of *Tp53* [225], indicating that p53-maintained genetic stability may be largely mediated by p21. However, p21 expression can also be induced independently of p53 [209]. Indeed, several studies have reported that an Ras/MAPK/ERK stimulus induces p21 expression independently of p53 [6, 226–228]. Therefore, both anti and pro-mitogenic pathways can induce the expression of the genomic guardian p21.

**Fig. 4:**
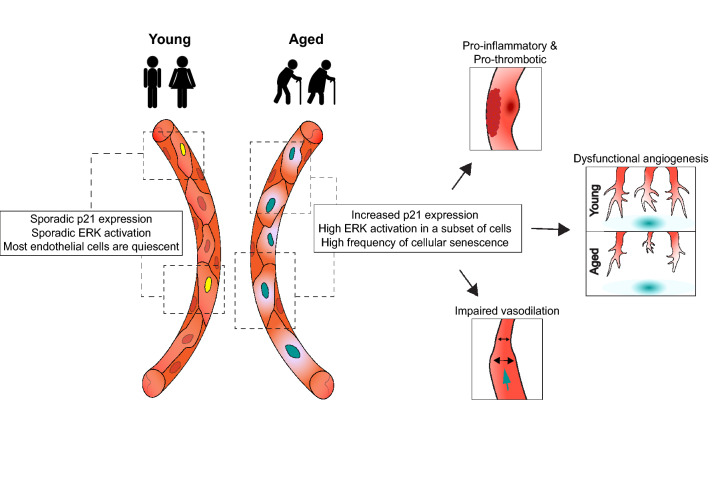
Consequences of ERK and p21 activation in aged blood vessels. In quiescent vessels of young individuals, p21 expression and transient ERK activation occur only sporadically in ECs as quiescence is maintained independently of p21. With progressing age, p21-positive cells having ERK constitutively activated accumulate in blood vessels. This senescent phenotype promotes a pro-inflammatory and pro-thrombotic environment in the endothelium, impairs vasodilation, and leads to dysfunctional angiogenesis

Aging is also a strong inducer of p21 expression (Fig. 4). For instance, a comparison between ECs obtained from young and aged human antecubital veins or brachial arteries revealed a 119% (veins) and 23% (arteries) increase in p21 expression with aging [216]. Similarly, the number of p21-positive ECs in the mouse aorta has been reported to be increased by approximately twofold in aged compared to young mice [219]. Of note, a significant baseline expression in normal blood vessels has been observed only of p21, while expression of the other common senescence/aging marker p16 has been demonstrated to be barely detectable in ECs from young adult mice and humans [216, 219, 229]. This correlates with data obtained from scRNAseq analyses of mouse lung ECs [168, 169]. The differential effects of transient and continued p21 expression in the growth arrest of ECs during developmental and tumor angiogenesis will be also interesting to explore. In most tip cells, the detected p21 expression [6] is likely only transient as the tip cell state has been suggested to be transient and highly dynamic [6, 56]. On the other hand, a high and persistent mitogenic stimulus, such as after deletion of *Dll4* or the Notch regulator *Rbpj*, results in the long term and sustained expression of p21 [6], which could lead to an irreversible cell cycle arrest or senescence. Currently, the role of Notch in EC senescence remains to be understood as both activation [230, 231] and inhibition [232] of Notch have been shown to promote a pro-senescent phenotype of ECs. However, Notch signaling exerts context and time-dependent effects in a growing vasculature which needs to be taken into account when interpreting these results. Interestingly, it has been reported that the increased expression of the Notch target gene *HES1* was essential for quiescent cells to be able to resume cell cycle in response to mitogenic stimuli and to prevent the senescence of human fibroblasts and tumor cells [171]. If a similar mechanism occurs in ECs, these results would suggest that quiescent and proliferating stalk ECs, which have higher levels of Notch activation [1, 30], would be more resistant to acquiring a senescent phenotype. On the other hand, tip cells having lower or inactive Notch signaling would be more prone to acquiring a senescent phenotype, if they remain in their tip cell position and therefore continuously express p21. Interestingly, besides expressing p21 and exiting cell-cycle, arrested tip cells also have some other senescent cell-like features, such as larger size than stalk cells, larger biosynthetic activity, and expression of secreted molecules, such as Esm1, Apln, Angpt2, and higher glycolytic activity [3, 41, 65, 233]. It is still to be determined if they also have other markers of senescence, such as higher senescence-associated beta-galactosidase activity or reactive oxygen species.

In contrast to angiogenic vessels, Dll4-signaling blockade of adult organs vessels seems to induce a transition from quiescence to proliferation [234], being yet unclear if hypermitogenic stimulus-associated senescence is also increased after this treatment. Similarly, endothelial-specific deletion of *Rbpj* in adult mice results in an increase in coronary vessel density and ERK1/2 phosphorylation; however, this leads to cardiac hypertrophy and heart failure [235].

Differences in senescence mechanisms between physiological angiogenesis, vascular homeostasis, and pathologic settings in young and aged tissues remain to be understood. Indeed, aging has been shown to affect tissues differently dependent on their exposure to stressors or organ-specific diseases and therapies [206]. Distinct organ vascular beds may also have marked differences in senescence frequencies and mechanisms. In regenerating tissues, the presence of senescent cells even appears to be crucial for adequate healing of the injury [176, 236], suggesting that not only the presence but also the clearance of senescent cells is necessary for regeneration. Indeed, effective immunosurveillance and clearance of senescent cells appear to be a critical factor in limb regeneration [205]. In addition, therapeutic drugs which specifically target senescent cells (senolytics) could be applied to improve angiogenesis and regeneration [206]. However, more research is necessary as it has been reported that removal of senescent cells both improves [237] or impairs [176, 236] tissue regeneration. A recent study even suggests that p16-positive ECs in the liver and other organs are essential for a healthy lifespan as their removal leads to a disruption of the blood–tissue barrier [238]. Collectively, these findings demonstrate the need for a better understanding of the molecular mechanisms controlling the reversible or irreversible induction of cellular arrest and how it can be modulated in therapeutic settings. The above findings also highlight the need for better genetic and imaging tools to probe cardiovascular proliferation and senescence with higher cellular and temporal resolution in health and disease.

## Vascular anomalies caused by abnormal mitogenic stimulation

Aberrant cell proliferation as a result of genetic mutations leads to uncontrolled tissue growth and is a key feature of cancer [44]. Pathologies associated with dysregulated EC proliferation or differentiation are summarized under the umbrella term “vascular anomalies”, which is further divided into benign and malign vascular tumors and vascular malformations [11, 12, 239]. One of the main differences between a tumor and a malformation is that malformations are mainly caused by congenital or sporadic developmental abnormalities present at birth, while vascular tumors arise in adults [11, 12, 239, 240]. One exception from this rule is the benign hemangioma which is a skin birthmark formed by excessive blood vessel growth. Although the molecular basis for it is still unknown and likely multifactorial, it has been suggested that this benign vascular tumor occurs due to congenital defects [12]. On the other hand, malignant vascular tumors, named angiosarcomas, arise spontaneously and later in life, due to a later sporadic event or as result from exposure to radiation or toxic chemicals [240]. Due to their relatively low occurrence and multiple causes and forms, treatment options, especially for life-threatening vascular anomalies, are mostly limited to currently used anti-angiogenic drugs or surgical intervention, and additional research is required to identify specific targetable mechanisms [11, 240, 241]. Although they occur sporadically, familiar forms of vascular anomalies have been key to the discovery of the pathophysiological causes. Inherited loss-of-function genetic mutations have been found in bone morphogenic protein (BMP) and transforming growth factor-β (TGF-β) receptor genes in hereditary hemorrhagic telangiectasia (HHT) [242–246]; several *CCM* genes causing cerebral cavernous malformations [247–249], glomulin in inherited glomuvenous malformations (GVM) [250], *RASA1* in capillary malformation–arteriovenous malformations (CM–AVM) [251], and weak germline gain-of-function (GOF) mutations in the *TIE2/TEK* gene appear to be the main genetic cause of capillary–venous malformations [252, 253].

With the advent of next-generation sequencing, it has been possible to identify also the genetic causes of sporadic and mosaic vascular anomalies [11]. For instance, it was shown that a somatic mutation in the *TIE2/TEK* gene occurs in 60% of these vascular anomalies resulting in a stronger increase in receptor activity compared to the inherited TIE2 mutations [254]. Germline mutations cannot cause a significant increase in receptor activity; otherwise, it would not enable vascular and embryo development and the subsequent germline transmission. On the other hand, somatic mutations occurring sporadically in vascular progenitor cells need to cause a stronger change in protein activity to induce a significant selective or competitive advantage of the mutant cells and hence a significant malformation. For this reason, sporadic malformations can be even more aggressive and deleterious than germline malformations.

**Fig. 5:**
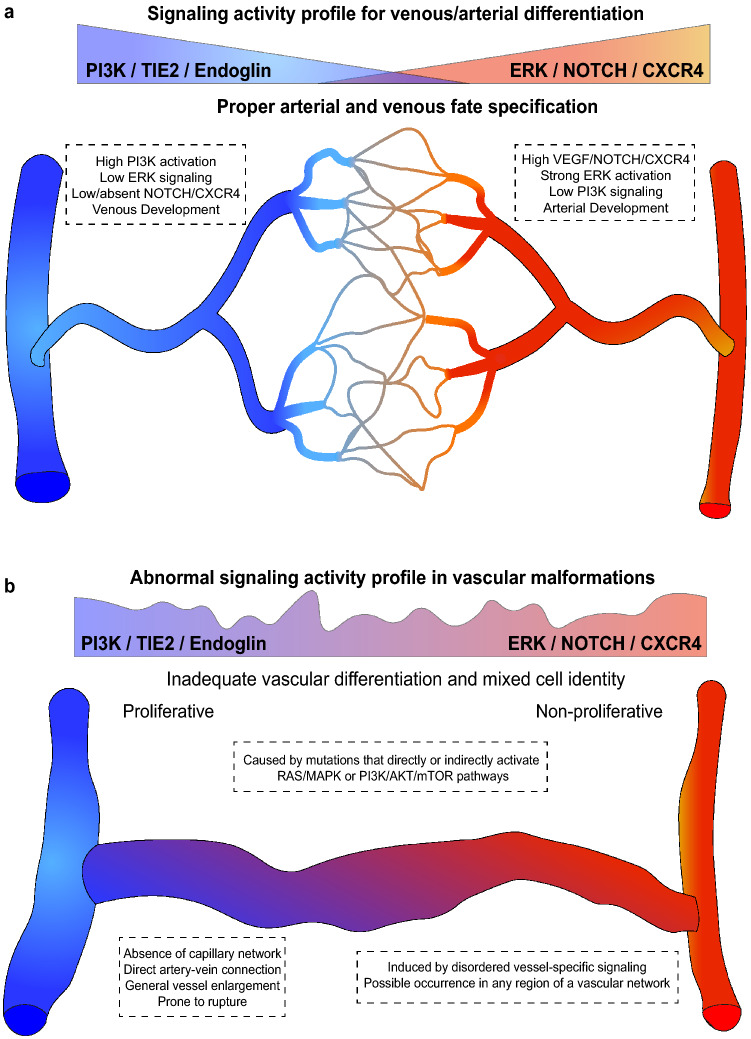
Abnormal endothelial signaling driving vascular malformations. **a** Artery–vein specification is ensured by separate, counteracting arteriovenous signaling pathways. The adequate commitment to either the arterial or venous fate allows the proper formation of a hierarchical network of blood vessels with a capillary plexus between the arterial and venous vessels. **b** Abnormal endothelial signaling results in improper AV differentiation and the formation of a direct artery–vein connection without an intermediate capillary network. These aberrant arteriovenous connections lead to vascular dysfunction and are prone to rupture

Most vascular malformations inducing mutations identified so far result in the direct or indirect activation of the PI3K/AKT/mTOR or RAS/MAPK/ERK pathways [11] (Fig. 5a, b). This is no surprise as these two pathways belong to the most dysregulated pathways found in human cancers and are highly relevant for the control of cell growth [255, 256]. Both pathways can be activated by various growth factor receptor subtypes including receptor tyrosine kinases (RTKs) or G protein-coupled receptors (GPCRs) [63, 255] and by the transactivation of RTKs by GPCRs [257–260]. However, even though the activation of PI3K signaling often results in proliferative venous malformations, the increase in RAS/MAPK/ERK activity often induces non-proliferative arterial-venous malformations which is in agreement with the bell-shaped response to mitogenic/VEGF/ERK signaling [6] discussed above.

Gain-of-function mutations of the RTK TIE2/TEK, which also leads to the activation of PI3K signaling, are responsible for around half of venous malformations in humans [261]. Rapamycin, an inhibitor of the PI3K/AKT/mTOR pathway, significantly improved the quality of life and reduced bleeding, lesion size, intravascular coagulopathy, and functional and esthetic impairment of patients suffering from TIE2-mutated venous malformations [262]. Importantly, about 30% of patients suffering from venous malformations without a *TIE2/TEK* mutation have been discovered to contain a gain-of-function mutation in the *PI3KCA* gene, showing the central role of this pathway in this type of vascular anomalies [263, 264]. Recently, it was demonstrated that pharmacological inhibition of PI3K also prevents VMs in a mouse model of HHT caused by *Alk1* inactivation or BMP9/10 ligand blockade [265]. Similarly, VMs induced by EC-specific deletion of *Eng* (encoding for endoglin) were partly normalized by blocking PI3K [266], highlighting the role of the BMP/TGF-β pathway in the formation of VMs. Notably, genetic deletion of *Eng* only in veins and capillaries but not in arteries has been shown to be sufficient for the development of malformations in the vasculature [267]. Furthermore, endothelial-specific activation of the PI3K pathway induced by deletion of *Pten*, an inhibitor of PI3K activation, has been shown to promote vascular hyperplasia in vivo [268]. For additional information on the role of the PI3K pathway in vascular malformations, the reader is directed to recent review articles [252, 261, 269, 270].

In contrast to the PI3K/AKT/mTOR pathway, observed mutations in the RAS/MAPK pathway generally induce arteriovenous malformations (AVMs). AVMs are morphologically characterized by the absence of a functional capillary network that links arteries and veins, resulting in a direct arterial-venous connection (Fig. 5a, b). In cerebral AVMs, for instance, most of the pathology arises from abnormal arterial-venous direct connections that are prone to leakage or even rupture leading to hemorrhage and the subsequent progressive development of the disease [271]. Several signaling mechanisms have been identified to influence arterial-venous fate decisions during development [272]. Early in vascular development, ECs begin to differentiate towards an arterial or venous lineage even in the absence of blood flow [75, 76, 273–275]. Interestingly, VEGF and downstream ERK1/2 activation seem to be strongly activated in cells directed towards arterial fate, while PI3K activation and ERK1/2 suppression drive venous fate decision [274–278] (Fig. 5a). However, contradictory results have been obtained in a recent study conducted in zebrafish where inhibition of ERK1/2 activity prevented sprouting angiogenesis but not initial artery differentiation [64]. Still, it is generally considered that VEGF, not necessarily ERK activity, is a key factor for arterial differentiation [64, 279].

Besides regulating MAPK/ERK activity, VEGF has also been shown to be responsible for the activation of Notch, a major pathway essential for arterial development and differentiation [30]. Indeed, VEGF induces the expression of the Notch ligand Dll4, which levels are the highest in arterial and sprouting tip ECs [1, 280] and lower in capillaries and absent in veins [79]. Absence of Dll4-Notch signaling results in the specific loss of the arterial genetic program [74–76, 274, 281]. Conversely, increased levels of Dll4 in developing embryos lead to ectopic induction of arterial markers in venous ECs [282]. It is, however, curious that Notch induces arteriogenesis and, at the same time, blocks ERK signaling, whereas VEGF induces ERK signaling and Notch. It is therefore likely that other yet unidentified factors, controlled by Notch and not ERK signaling, play an important role.

Recently, data obtained in the mouse retina and zebrafish embryo angiogenesis models indicate that high Cxcr4/Notch expressing tip ECs migrate against the blood flow to form arteries, thereby providing a more dynamic mechanistic insight into the role of Cxcr4 and Dll4-Notch signaling in arterialization [283, 284]. A similar genetic signature and mechanism were identified in capillary pre-arterial ECs in developing mouse hearts by scRNAseq and genetic lineage tracing analysis [285]. Genetic deletion experiments in developing coronary vessels confirmed the importance of Dll4-Notch signaling for the formation of coronary arteries [286, 287]. Collectively, these studies suggest that pre-arterial capillaries contain pre-determined endothelial cells, having an arterial signaling and genetic profile consisting of high VEGF/Notch/Cxcr4 activity that commits them to form arteries.

These findings obtained in distinct angiogenesis model systems are very important to understand the consequence of mutations causing human AVMs. As an example, a recent study has shown that ECs from human brain AVMs have increased Ras and Notch activity, suggesting that the underlying cause of AVMs may be excessive arterialization of blood vessels [288]. Constitutive activation of Notch signaling in the mouse postnatal veins and capillaries also leads to AVMs [289] (Fig. 5b). A recent report has shown that Notch activation results in upregulation of the arterial marker Connexin 37 (Cx37) and the cell cycle inhibitor p27, inducing cell cycle arrest and thereby halting endothelial proliferation [186]. However, *Cx37* KO and *Cdkn1b* KO mice are viable and reach adulthood without any major arteriogenesis or vascular defects [120, 290], suggesting the existence of alternative Notch-dependent mechanisms. Still, it remains to be fully investigated if the cell cycle arrest during arterial cell differentiation is merely a consequence or causative of arterialization and which are the underlying mechanisms. A recent study showed that overexpression of CoupTFII can impair arterialization by simply inducing cell cycle genes in pre-arterial ECs [285].

It is clear from all the above studies that pathways commonly associated with the regulation of cell proliferation can also regulate cell differentiation, depending on the dose of signaling and cellular context. It is plausible that MAPK activating mutations in AVMs cause more differentiation than proliferation defects. Sporadic activating mutations in RAS pathway genes or its regulators are very frequent and cause RASopathies affecting more than 1 in 1000 people. RASopathies are frequently associated with several tissue development abnormalities, including vascular malformations [291]. However, rarer mutations induce vascular anomalies not associated with other tissue abnormalities. This is the case for loss-of-function mutations in *Rasa1*, a gene coding for an important negative regulator of Ras activity. Mutations in this gene have been discovered in patients suffering from CM–AVM and Parks-Weber Syndrome [251, 292, 293]. Yet, data in these studies are still relatively descriptive and are still unclear how the reported mutations affect EC signaling or growth. Somatic activating mutations in the gene *GNAQ,* which encodes for the G protein subunit G_q/11_ inducing MAPK activation, have been associated also with vascular anomalies such as capillary malformations and Sturge–Weber syndrome [11, 294]. When transfected into cells, the mutated variant of *GNAQ*, however, only induced a mild activation of MAPK signaling, and no effect on endothelial proliferation was described [294]. Although activating mutations of HRAS can result in increased ERK phosphorylation and uncontrolled EC proliferation, resulting in cerebrovascular malformations in a mouse model [295], KRAS mutations observed in human brain malformations did influence ERK activation and angiogenic marker expression but not EC proliferation [288].

Besides germline and congenital vascular anomalies, present at birth, there are also another category of vascular anomalies named as angiosarcomas. These usually develop much later in life as a result of exposure to toxic chemicals, radiation therapy or damaging UV light [296]. Angiosarcomas are frequently found in the head and neck vessels and less frequently in breast, liver, and heart vessels. They are usually highly proliferative and can metastasize to other organs, resulting in significant morbidity and mortality [297]. Treatment is usually non-specific and aimed at inhibiting general cell proliferation pathways. Recent developments in DNA sequencing have uncovered a range of genetic mutations in genes important for the regulation of angiogenesis. A recent review article has listed a collection of studies reporting genes and their occurrences of mutations in angiosarcoma tumor tissues, such as *KDR* (the gene was mutated in 5.9–7% of analyzed tumor samples), *PLCG1* (2.9–20%), *PTPRB* (17.6–25.6%), *KRAS* (2.6–2.9%), *HRAS* (5.5–11.8%), *NRAS* (5.5–5.9%), *BRAF* (11.8%), *MAPK1* (2.9%), *NFL1* (2.6–2.9%), *PI3KCA* (2.6–16.7%), *TP53* (4–35%), *CIC* (2–6%), *ROS1* (3%), and *CDKN2A* (26.5%), as summarized in [296]. Several of these mutations found in angiosarcomas directly or indirectly affect the MAPK pathway [298]. Furthermore, frequent amplifications of *MYC* or *VEGFR3* were detected in radiation-induced angiosarcomas [299, 300]. This information is being used to develop more targeted pharmacological approaches against angiosarcoma [296, 297]. Interestingly, through genetic deletion experiments in mice, a link between angiosarcomas and *Cdkn* genes was suggested [122]. Specifically, in mice older than 28 weeks containing various combinations of global *Cdkn1a/Cdkn1b* deletion in addition to a Cdk4 mutation (i.e., hetero- or homozygous deletion/mutation), between 9 and 56% of all spontaneously formed tumors were angiosarcomas [122, 161]. Furthermore, 23% of all tumors in *Cdkn2a*-null mice have been shown to be angiosarcomas [156]. Collectively, this proves the significance of *Cdkn* gene function in maintaining endothelial homeostasis and quiescence, and demonstrates that loss-of-function mutations or deletions within these genes or pathways can be potent drivers of disease.

## Conclusion

The concept that ECs in a growing vessel have different sprouting and proliferative abilities has been introduced 15 years ago [55], but it took many more years of intense research by several groups to identify the most basic molecular mechanisms involved in the differentiation of tip and stalk cells, a process of high relevance for angiogenesis. It is however interesting to notice that the large majority of these studies were conducted in zebrafish embryos or in the mouse retina angiogenesis system, and little is known about endothelial heterogeneity and the dynamic behavior of tip and stalk cells in other organ vessels. The field has in general assumed that the most basic molecular and cellular mechanisms are highly conserved and should be involved in all events of sprouting angiogenesis even in other organs and pathological contexts, but more experimental evidence will be needed to confirm this. This is of special relevance, since existing evidence points towards significant discrepancies between zebrafish intersomitic vessel sprouting and mouse retina angiogenesis sprouting. The most important of it is the fact that the majority of mouse retina endothelial tip cells do not proliferate [6, 55], whereas most zebrafish intersegmental vessel tip cells proliferate, while they migrate [80]. With the advent of scRNAseq, it is now possible to discover signatures of sprouting and proliferative cells in virtually any developmental context and organ. Even though the field has initially focused in scRNAseq analysis of wild-type adult organ vessels [7, 169, 301–303], the number of different ongoing scRNAseq studies and developmental contexts analyzed is rapidly increasing and already some studies provided an unprecedented level of cellular resolution on the molecular mechanisms driving developmental angiogenesis [285, 304]. The combination of targeted functional genetics with scOMICS will certainly change our understanding of vascular cell heterogeneity and the process of angiogenesis.

Modulating the already identified mechanisms of angiogenesis to induce or inhibit functional blood vessel formation in ischemic vascular disease or in cancer has yet to show a very significant clinical benefit [102, 108, 305]. The most successful and widespread clinical use of anti-angiogenesis has been in the treatment of ocular disease [22, 306]. Single pathway targeting anti-angiogenesis therapies in cancer face the problem of resistance or alternative modes of vessel growth, and combinatorial targeting seems to be the best approach [23, 41], even though, in most cases, tumors can continue to grow, or even become more metastatic in the absence of angiogenesis [307, 308]. Vascular normalization for effective chemotherapeutics delivery is another use of anti-angiogenesis, but so far with relatively limited use [23].

It is clear that is far easier to block angiogenesis, than to promote it. Waking up a dormant vasculature and inducing effective and functional vascular growth is far more challenging but also of great potential clinical benefit. Simply promoting EC proliferation may not be the best way to achieve effective tissue vascularization and healing. Recently, administration of Cxcl12 to injured hearts, a chemokine that binds the receptor Cxcr4 and is key for EC chemotactic migration, has shown a significant effect on the development of collateral arteries in ischemic heart disease [309]. This study and others have shown that to effectively induce angiogenesis in the clinics, we will have to understand and mimic as much as possible the mechanisms used by developing tissues. Achieving the proper spatiotemporal mitogenic and sprouting balance with pharmacological compounds will be key [47]. But first, we need to understand what regulates the bell-shaped response of endothelial cells to mitogenic stimulation and find ways to achieve a therapeutic equilibrium to achieve maximal sprouting and proliferation.

It is interesting to note that the vast majority of mutations detected in patients suffering from vascular malformations or angiosarcomas affect relatively few genes or key downstream signaling mechanisms. Recapitulating or modeling the timing and mosaic spatial occurrence of these human mutations in lower organisms will be key to analyze and understand the cascade of underlying signaling and cellular events triggered by a given mutation.

Progress in the understanding and treatment of cancer, cardiovascular disease, and congenital vascular malformations will require the collaboration and coordinated efforts of developmental biologists, bioinformaticians, and clinicians. Different backgrounds, experimental approaches, and expertises need to be integrated to effectively uncover mechanisms of clinical relevance and develop new treatments.
